# The impact of weight loss related to risk of new-onset atrial fibrillation in patients with type 2 diabetes mellitus treated with sodium–glucose cotransporter 2 inhibitor

**DOI:** 10.1186/s12933-021-01285-8

**Published:** 2021-04-30

**Authors:** Yi-Hsin Chan, Shao-Wei Chen, Tze-Fan Chao, Yi-Wei Kao, Chien-Ying Huang, Pao-Hsien Chu

**Affiliations:** 1 The Cardiovascular Department, Chang Gung Memorial Hospital, Linkou, Taoyuan 33305 Taiwan; 2 College of Medicine, Chang Gung University, Taoyuan, 33302 Taiwan; 3 Microscopy Core Laboratory, Chang Gung Memorial Hospital, Linkou, Taoyuan Taiwan; 4 Division of Thoracic and Cardiovascular Surgery, Department of Surgery, Linkou Medical Center, Chang Gung Memorial Hospital, Chang Gung University, Taoyuan City, Taiwan; 5 Center for Big Data Analytics and Statistics, Linkou Medical Center, Chang Gung Memorial Hospital, Taoyuan City, Taiwan; 6 Division of Cardiology, Department of Medicine, Taipei Veterans General Hospital, Taipei, Taiwan; 7 Institute of Clinical Medicine, Cardiovascular Research Center, National Yang Ming Chiao Tung University, Taipei, Taiwan; 8 Graduate Institute of Business Administration, College of Management, Fu Jen Catholic University, Taipei, Taiwan

**Keywords:** Atrial fibrillation, Type 2 diabetes mellitus, Sodium–glucose cotransporter-2 inhibitor, Heart failure, Obesity

## Abstract

**Background:**

Sodium–glucose cotransporter 2 inhibitor (SGLT2i) use reduces body weight (BW) in patients with type 2 diabetes mellitus (T2DM). Obesity and T2DM are strong risk factors of new-onset atrial fibrillation (AF). However, whether BW loss following SGLT2i treatment reduces AF risk in patients with T2DM remains unclear.

**Methods:**

We used a medical database from a multicenter health care provider in Taiwan, which included 10,237 patients with T2DM, from June 1, 2016 to December 31, 2018, whose BW data at baseline and at 12 weeks of SGLT2i treatment were available. Patients were followed up from the drug index date until the occurrence of new-onset AF, discontinuation of the SGLT2i, or the end of the study period, whichever occurred first.

**Results:**

The patients’ baseline body mass index (BMI) was 28.08 $\pm$ 4.88 kg/m^2^. SGLT2i treatment was associated with a BW loss of 1.35 $\pm$ 3.28 kg (1.78%$\pm$ 4.47%). There were 37.4%, 47.0%, and 15.6% of patients experienced no-BW loss (n = 3832), BW loss 0.0–4.9% (n = 4814), and $\ge$ 5.0% (n = 1591) following SGLT2i treatment, respectively. Compared with patients with baseline BMI < 23 kg/m^2^, AF risk significantly increased in patients with baseline BMI $\ge$ 27.5 kg/m^2^ (*P* for trend = 0.015). Compared with those without BW loss after SGLT2i treatment, AF risk significantly decreased with a BW loss of $\ge$ 5.0% (adjusted hazard ratios [95% confidence intervals]: 0.39[0.22–0.68]). Use of diuretics, old age, high-dose SGLT2i, higher estimated glomerular filtration rate, and baseline BMI were independent factors associated with a BW loss of $\ge$ 5.0% following SGLT2i initiation. By contrast, neither baseline BMI nor BW loss after SGLT2i treatment predicted major cardiovascular adverse events or heart failure hospitalization risk (*P* for trend > 0.05).

**Conclusion:**

BW loss of ≥ 5.0% following SGLT2i treatment was associated with a lower risk of new-onset AF in patients with T2DM in real-world practice.

## Background

Atrial fibrillation (AF), the most common sustained cardiac arrhythmia worldwide, is associated with a fivefold increased ischemic stroke risk and twofold increased mortality risk [1]. Diabetes mellitus (DM) is an independent risk factor for new-onset AF in the general population [2, 3]. Pathophysiological mechanisms, including atrial electrical, structural, neural remodeling, and glycemic fluctuations, may play a crucial role in increased AF risk in patients with DM [4]. Sodium–glucose cotransporter 2 inhibitors (SGLT2is) are a new class of antidiabetic drug that inhibits renal tubular sodium–glucose reabsorption without stimulating insulin release in patients with type 2 DM (T2DM) [5]. Large randomized placebo-controlled trials have concluded that SGLT2is (including canagliflozin, dapagliflozin, and empagliflozin) reduced the risk of major cardiovascular events, heart failure hospitalization, and stabilized renal function consistently in patients with T2DM with or without established cardiovascular diseases [6–8]. Furthermore, post hoc analysis of the DECLARE-TIMI 58 trial and a few real-world data indicated that the use of dapagliflozin and other SGLT2is was associated with a lower new-onset AF/atrial flutter risk than current standard care of antihyperglycemic agents in patients with T2DM [9, 10]. SGLT2is have multiple pleiotropic effects of glucose-independent and direct cardiac protection, including reduction in inflammation, oxidative stress, endothelial dysfunction, and left ventricular dysfunction, which may improve atrial remodeling and thus reduce AF risk [11, 12]. Long-term sustained weight loss through diet and physical activity modification can significant reduce AF burden and maintain sinus rhythm in obese individuals with AF or those who underwent post–catheter ablation for AF [11, 13]. Furthermore, SGLT2is directly cause body weight (BW) loss through glucose excretion (calorie loss); however, how BW loss affects new-onset AF risk in patients with T2DM remains unclear. Therefore, this study, by using a large real-world database of an Asian population with T2DM, evaluated whether BW loss due to SGLT2i treatment reduces the risk of new-onset AF.

## Methods

### Database

This retrospective observational study was approved by the Institutional Review Board of the Chang Gung Medical Foundation. It was based on data from the Chang Gung Research Database provided by Chang Gung Memorial Hospital (CGMH). The interpretation and conclusions contained herein do not represent the position of CGMH The CGMH Medical System is composed of two medical centers, two regional hospitals, and three district hospitals, with a total of 10,050 beds and approximately 280,000 admissions per year; it is currently the largest health care provider in Taiwan [14]. The advantage of the CGMH medical database detailed data on diagnoses, medications, interventions, laboratory examinations, and imaging are available for each patient [14]. The identification number of each patient is encrypted and de-identified using a consistent encryption procedure; therefore, the need for informed consent was waived for this study.

### Study design and outcome

**Fig. 1:**
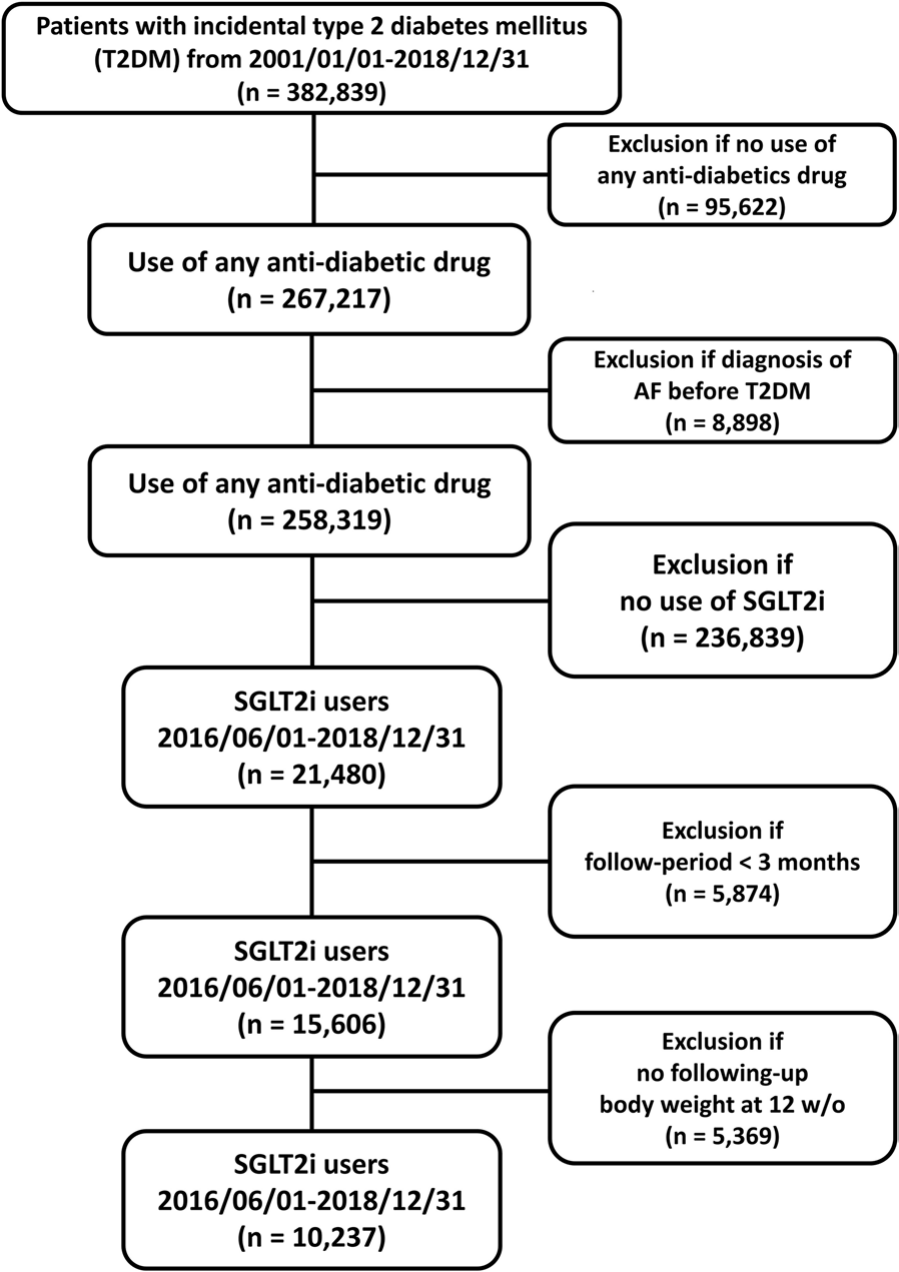
Enrollment of patients with type 2 diabetes mellitus (T2DM) treated with sodium–glucose cotransporter 2 inhibitors (SGLT2i). In total, 10,237 patients with T2DM without prevalent AF treated with SGLT2i were enrolled from June 1, 2016, to December 31, 2018. AF: atrial fibrillation; SGLT2i: sodium–glucose cotransporter 2 inhibitor; T2DM: type 2 diabetes mellitus

Figure 1 presents the study design and patient enrollment flowchart. The CGMH Research Database was retrospectively searched for patients aged $\ge$ 20 years in whom new-onset T2DM was diagnosed from January 1, 2001, to December 31, 2018 (n = 382,839). We excluded patients who did not use any antidiabetic drugs (n = 95,622) and who had a diagnosis of prevalent AF before T2DM diagnosis (n = 8,898). Among the remaining 258,319 patients, those who had a first prescription for a SGLT2i (approval date: June 1, 2016) were enrolled in the present study (n = 21,480). We included only patients with a follow-up period of > 3 months. We used the BW data nearest to the date of 12 weeks (3 months) after the drug index date as the following-up BW data after SGLT2i treatment, to calculate the change of BW following SGLT2i treatment ((following-up BW—baseline BW)/baseline BW (%)). Patients without BW data at baseline and at ~ 12 weeks of SGLT2i treatment were excluded. Finally, 10,237 SGLT2i users with paired BW data were considered for analysis. The study outcome was the diagnosis of new-onset AF (International Classification of Diseases, Ninth Revision, Clinical Modification [ICD-9-CM] code 427.31 from January 1, 2010, to December 31, 2015, and ICD-10-CM code I48 from January 1, 2016, to December 31, 2018) in at least one inpatient or outpatient department visit that occurred at least 12 weeks after the drug index date (i.e., the first date of a prescription for a SGLT2i after June 1, 2016). The follow-up period was defined as the period from the index date until the occurrence of new-onset AF, discontinuation of the index drug, mortality, last follow-up date in the CGMH Medical System, or the end of the study period (December 31, 2018), whichever occurred first.

### Covariates

Baseline characteristics referred to any claims record with the aforementioned diagnoses or medication codes prior to the drug index date. A history of any prescription medicine was confined to medications taken at least once within 3 months preceding the index date. Baseline laboratory data listed in Table 1 were based on the measurements performed within 1 year before the drug index date.

**Table 1:** Clinical characteristics of patients with type 2 diabetes treated with an SGLT2i stratified by baseline BMI

	Normal BMI < 23 (n = 1,203)	Overweight BMI 23.0–24.9 (n = 1,518)	Obese I BMI 25.0–27.4 (n = 2,465)	Obese II BMI 27.5–29.9 (n = 2,026)	Obese III BMI $\ge$ 30.0 (n = 3,025)	*P value (ANOVA)*
Clinical characteristics						
Diabetes duration (year)	8.5 ± 3.6	8.4 ± 3.7	8.5 ± 3.5	8.3 ± 3.6	7.6 ± 3.7	< 0.001
Age (year)	62.0 ± 10.7	61.1 ± 10.7	60.0 ± 10.7	58.9 ± 10.9	54.6 ± 12.1	< 0.001
Female	601 (50)	643 (42)	1042 (42)	768 (38)	1290 (43)	< 0.001
Ischemic heart etiology	89 (7)	123 (8)	215 (9)	194 (10)	194 (6)	0.001
Hypertension	630 (52)	878 (58)	1634 (66)	1437 (71)	2264 (75)	< 0.001
Dyslipidemia	799 (66)	1125 (74)	1883 (76)	1536 (77)	2318 (77)	< 0.001
Cerebral vascular accidents	53 (4)	75 (5)	111 (5)	90 (4)	110 (4)	0.273
Congestive heart failure	47 (4)	42 (3)	78 (3)	66 (3)	98 (3)	0.585
Chronic lung disease	26 (2)	29 (2)	42 (2)	36 (2)	89 (3)	0.011
Chronic liver disease	308 (26)	353 (23)	680 (28)	588 (29)	912 (30)	< 0.001
Chronic kidney disease	208 (17)	248 (16)	415 (17)	338 (17)	543 (18)	0.633
Peripheral artery disease	13 (1)	15 (1)	22 (1)	18 (1)	21 (1)	0.741
Gout	70 (6)	115 (8)	227 (9)	241 (12)	386 (13)	< 0.001
Malignancy	111 (9)	155 (10)	203 (8)	125 (6)	213 (7)	< 0.001
Vital sign						
Height (cm)	160.6 ± 12.2	161.8 ± 11.8	161.8 ± 10.9	162.6 ± 11.8	162.1 ± 14.2	< 0.001
Body weight (KG)	56.6 ± 7.4	63.8 ± 6.8	69.2 ± 7.6	76.5 ± 8.4	89.3 ± 14.3	< 0.001
BMI	21.4 ± 1.4	24.1 ± 0.6	26.2 ± 0.7	28.6 ± 0.7	33.6 ± 3.8	< 0.001
SBP (mmHg)	133.4 ± 20.8	136.5 ± 18.9	138.2 ± 20.0	140.2 ± 18.5	142.0 ± 19.2	< 0.001
DBP (mmHg)	73.7 ± 10.9	76.1 ± 11.1	77.2 ± 11.1	78.8 ± 11.4	80.8 ± 11.4	< 0.001
HR (bpm)	85.0 ± 13.5	84.0 ± 13.3	84.0 ± 13.3	84.4 ± 13.1	85.8 ± 13.7	< 0.001
Baseline laboratory data						
HbA1c (%)	9.1 ± 1.8	8.8 ± 1.6	8.8 ± 1.6	8.8 ± 1.6	8.7 ± 1.6	< 0.001
eGFR (ml/min/m^2^)	97.1 ± 33.9	93.6 ± 29.1	93.0 ± 30.3	91.7 ± 28.2	96.1 ± 29.4	< 0.001
ALT (U/L)	28.3 ± 27.3	29.4 ± 31.3	33.3 ± 64.0	35.0 ± 33.6	40.8 ± 32.3	< 0.001
Triglycerides (mg/dL)	137.6 ± 135.0	157.9 ± 141.0	180.6 ± 230.6	199.7 ± 323.8	198.1 ± 187.9	< 0.001
LDL (mg/dL)	94.9 ± 30.8	93.3 ± 29.9	92.2 ± 30.5	93.7 ± 31.2	94.4 ± 29.2	0.050
HDL (mg/d)	48.3 ± 14.1	45.1 ± 11.1	43.7 ± 10.9	42.9 ± 10.4	42.4 ± 10.3	< 0.001
Baseline medications						
Anti-platelet agent	336 (28)	494 (33)	814 (33)	689 (34)	958 (32)	0.006
Statin	653 (54)	928 (61)	1566 (64)	1298 (64)	1880 (62)	< 0.001
Non-dihydropyridine CCB	52 (4)	67 (4)	106 (4)	131 (6)	180 (6)	0.001
Dihydropyridine CCB	135 (11)	210 (14)	387 (16)	330 (16)	554 (18)	< 0.001
Beta-blocker	278 (23)	421 (28)	747 (30)	708 (35)	1082 (36)	< 0.001
ACEI or ARB or ARNI	527 (44)	777 (51)	1440 (58)	1288 (64)	2081 (69)	< 0.001
MRA	30 (2)	20 (1)	64 (3)	57 (3)	95 (3)	0.008
Loop diuretics	72 (6)	76 (5)	156 (6)	120 (6)	237 (8)	0.003
Thiazides	4 (0)	7 (0)	13 (1)	8 (0)	33 (1)	0.005
Nitrate	61 (5)	68 (4)	150 (6)	141 (7)	178 (6)	0.023
Digoxin	14 (1)	10 (1)	21 (1)	13 (1)	21 (1)	0.469
Anti-diabetic agent						
SU	833 (69)	1070 (70)	1698 (69)	1340 (66)	1968 (65)	0.001
Metformin	1061 (88)	1357 (89)	2253 (91)	1850 (91)	2762 (91)	0.004
Glinide	61 (5)	39 (3)	85 (3)	61 (3)	82 (3)	0.001
DPP4i	655 (54)	772 (51)	1229 (50)	981 (48)	1356 (45)	< 0.001
Glitazone	263 (22)	351 (23)	594 (24)	564 (28)	803 (27)	< 0.001
Acarbose	297 (25)	329 (22)	518 (21)	406 (20)	565 (19)	< 0.001
Insulin	262 (22)	258 (17)	415 (17)	345 (17)	478 (16)	< 0.001
GLP1 agonist	3 (0)	8 (1)	14 (1)	10 (0)	45 (1)	< 0.001

ACEI: angiotensin-converting enzyme inhibitor; ALT: alanine aminotransferase; ARB: angiotensin receptor blocker; ARNI: angiotensin receptor-neprilysin inhibitor; BMI: body mass index; CCB: calcium channel blocker; DBP: diastolic blood pressure; DPP4i: dipeptidyl peptidase-4 inhibitor; eGFR: estimated glomerular filtration rate; GLP1: glucagon-like peptide 1; HBA1c: hemoglobin A1c; HDL: high-density lipoprotein; HR: heart rate; LDL: low-density lipoprotein; MRA: mineralocorticoid receptor antagonist; SBP: systolic blood pressure; SGLT2i: sodium–glucose co-transporter-2 inhibitor; SU: sulfonylurea

Data are expressed as mean ± standard deviation or number (%)

### Statistical analysis

Data are presented as mean ± standard deviation for continuous variables and as proportions for categorical variables. Analysis of variance was used to compare differences in continuous variables, and χ^2^ test was used to compare the differences in nominal variables. Crude incidence rates were computed as the total number of study outcomes during the follow-up time divided by person-years at risk. Kaplan–Meier method and multivariate Cox proportional hazards regression were used to compare the risk of events in patients with T2DM across different categories based on baseline body mass index (BMI) or BW loss after SGLT2i treatment. Statistical significance was set as *P* < 0.05. All analyses were conducted using SAS (version 9.2; SAS Institute, Cary, NC, USA).

## Results

### Baseline characteristics of ‘baseline BMI’ categories

The mean follow-up period was 1.5 $\pm$ 0.6 years. Of 10,237 patients, 5492 (54%), 4739 (46%), and 6 (0%) received empagliflozin, dapagliflozin, and canagliflozin, respectively. The mean age and baseline BMI for the study cohort were 58.6 $\pm$ 11.5 years and 28.1 $\pm$ 4.9 kg/m^2^, respectively. We stratified the patients based on their BMI into the following groups: normal (BMI < 23.0 kg/m^2^; n = 1203), overweight (BMI: 23.0–24.9 kg/m^2^; n = 1518), obese I (BMI: 25.0–27.4 kg/m^2^; n = 2465), obese II (BMI: 27.5–29.9 kg/m^2^; n = 2026), and obese III (BMI: $\ge$ 30.0 kg/m^2^; n = 3025) subgroups, modified from the WHO Asian BMI classifications [15]. Table 1 summarizes the clinical characteristics of patients with AF stratified by BMI. In general, patients with a higher baseline BMI were younger and predominantly female and had a higher prevalence of hypertension, dyslipidemia, and chronic liver disease. Moreover, a higher percentage of them received antiplatelet agents, beta-blockers, angiotensin-converting-enzyme inhibitors (ACEIs)/angiotensin II receptor antagonists (ARBs), and statins, and a lower percentage of them received insulin or oral hypoglycemic agents (*P* < 0.0001).

### Baseline characteristics of ‘body weight loss’ categories

Overall, a BW loss of 1.35 $\pm$ 3.28 kg (− 1.78% $\pm$ 4.47%) was noted in the study patients after 12 weeks of SGLT2i treatment. Patients were divided into three groups according to the amount of BW loss: No BW loss (n = 3832) and BW loss of 0.0–5.0% (n = 4814), and $\ge$ 5.0% (n = 1591). We used the cutoff value of 5% loss in BW because clinically significant BW loss was defined as those achieving ≥ 5% BW loss from baseline according to previous literatures [16, 17] Table 2 summarizes the clinical characteristics of patients with AF stratified by the amount of BW loss. In general, patients with a BW loss > 5% were older; had a higher prevalence of female in gender, ischemic heart disease, congestive heart failure; and had a higher baseline BMI but a lower HbA1c. Moreover, a higher percentage of them received antiplatelet agent, loop diuretics, and nitrate, but a lower percentage of them received insulin (*P* < 0.001).

**Table 2:** Clinical characteristics of patients with type 2 diabetes treated with an SGLT2i stratified by changes in body weight (BW)

	No BW loss (n = 3,832)	BW loss 0.0–5.0% (n = 4,814)	BW loss $\ge$ 5.0% (n = 1,591)	*P value (ANOVA)*
Clinical characteristics				
Diabetes duration (year)	8.1 ± 3.6	8.3 ± 3.6	8.1 ± 3.7	0.004
Age (year)	58.2 ± 11.6	58.4 ± 11.3	60.0 ± 11.8	< 0.001
Female	1610 (42)	2009 (42)	725 (46)	0.023
Ischemic heart etiology	313 (8)	352 (7)	150 (9)	0.022
Hypertension	2564 (67)	3231 (67)	1048 (66)	0.654
Dyslipidemia	2827 (74)	3734 (78)	1127 (71)	< 0.001
Cerebral vascular accidents	186 (5)	174 (4)	79 (5)	0.006
Congestive heart failure	150 (4)	108 (2)	73 (5)	< 0.001
Chronic lung disease	85 (2)	98 (2)	39 (2)	0.593
Chronic liver disease	1066 (28)	1374 (29)	401 (25)	0.036
Chronic kidney disease	711 (19)	793 (16)	248 (16)	0.008
Peripheral artery disease	32 (1)	40 (1)	17 (1)	0.648
Gout	409 (11)	489 (10)	141 (9)	0.132
Malignancy	319 (8)	361 (7)	127 (8)	0.363
Vital sign				
Height (cm)	162.1 ± 12.1	162.3 ± 12.0	160.5 ± 14.2	< 0.001
Body weight (KG)	73.3 ± 15.2	75.3 ± 15.0	73.5 ± 15.1	< 0.001
BMI (kg/m^2^)	27.6 ± 4.7	28.3 ± 4.7	28.1 ± 4.8	< 0.001
Body weight loss (KG)	1.4 ± 2.6	− 2.0 ± 1.0	− 5.9 ± 3.1	< 0.001
SBP (mmHg)	138.5 ± 20.2	139.1 ± 19.2	139.1 ± 19.6	0.324
DBP (mmHg)	78.0 ± 12.1	78.1 ± 11.3	77.7 ± 11.6	0.403
HR (bpm)	84.8 ± 13.5	84.6 ± 13.1	84.8 ± 13.4	0.696
Baseline laboratory data				
HbA1c (%)	9.0 ± 1.8	8.7 ± 1.5	8.8 ± 1.6	< 0.001
eGFR (ml/min/m^2^)	93.8 ± 31.7	94.7 ± 28.3	94.0 ± 30.3	0.391
ALT (U/L)	34.5 ± 33.7	34.8 ± 26.8	34.8 ± 80.6	0.915
Triglycerides (mg/dL)	190.6 ± 247.9	178.5 ± 222.6	166.2 ± 142.4	0.001
LDL (mg/dL)	95.1 ± 32.2	92.5 ± 28.8	93.6 ± 29.3	< 0.001
HDL (mg/d)	43.6 ± 11.3	44.0 ± 11.0	44.7 ± 11.6	0.003
Baseline medications				
Anti-platelet agent	1192 (31)	1541 (32)	558 (35)	0.017
Statin	2286 (60)	3079 (64)	960 (60)	< 0.001
Non-dihydropyridine CCB	210 (5)	232 (4)	94 (6)	0.166
Dihydropyridine CCB	616 (16)	760 (16)	230 (14)	0.318
Beta-blocker	1238 (32)	1488 (32)	510 (32)	0.350
ACEI or ARB or ARNI	203 (60)	2896 (62)	909 (57)	0.074
MRA	117 (3)	99 (2)	50 (3)	0.005
Loop diuretics	275 (7)	254 (5)	132 (8)	< 0.001
Thiazides	22 (1)	31 (1)	12 (1)	0.745
Nitrate	245 (6)	247 (5)	106 (7)	0.014
Digoxin	41 (1)	30 (1)	8 (1)	0.025
Anti-diabetic agent				
SU	2562 (67)	3286 (68)	1061 (67)	0.292
Metformin	3419 (89)	4440 (92)	1424 (90)	< 0.001
Glinide	123 (3)	155 (3)	50 (3)	0.988
DPP4i	1826 (48)	2409 (50)	758 (48)	0.054
Glitazone	941 (25)	1235 (26)	399 (25)	0.504
Acarbose	802 (21)	976 (20)	337 (21)	0.647
Insulin	798 (21)	721 (15)	239 (15)	< 0.001
GLP1 agonist	30 (1)	36 (1)	14 (1)	0.874

Data are expressed as mean ± standard deviation or number (%)

ACEI: angiotensin-converting enzyme inhibitor; ALT: alanine aminotransferase; ARB: angiotensin receptor blocker; ARNI: angiotensin receptor-neprilysin inhibitor; BMI: body mass index; BWG: body weight gain; BWL: body weight loss; CCB: calcium channel blocker; DBP: diastolic blood pressure; DPP4i: dipeptidyl peptidase-4 inhibitor; eGFR: estimated glomerular filtration rate; GLP1: glucagon-like peptide 1; HBA1c: hemoglobin A1c; HDL: high-density lipoprotein; HR: heart rate; LDL: low-density lipoprotein; MRA: mineralocorticoid receptor antagonist; SBP: systolic blood pressure; SGLT2i: sodium–glucose co-transporter-2 inhibitor; SU: sulfonylurea

### Long-term body weight trajectories across study groups

**Fig. 2:**
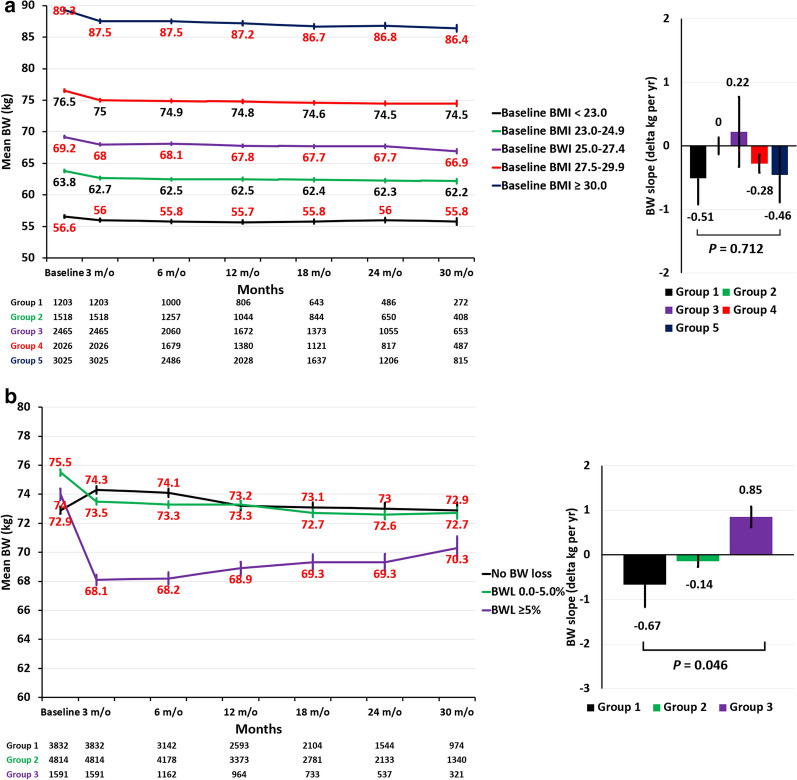
Long-term body weight (BW) trajectories across study groups. The log-term BW trajectories for the five study groups of different baseline BMI categories receiving SGLT2i treatment remained stable from 3 months onward in all study groups, and there is no difference of the BW slope across 5 study groups (*P*: 0.712) (**a**). The respective mean (SE) BW changes from baseline to the 3 months following-up in these 3 groups of different BW-loss categories following SGLT2i treatment were 1.4 (0.04), − 2.1 (0.14), and − 5.9 (0.08) kg. The mean (SE) BW slope from 3 months until the last available measurements among participants receiving SGLT2i was − 0.67 (− 0.50), − 0.14 (− 0.13), and 0.85 (0.23) kg/year in these 3 groups. There is significant difference of the BW slope across 3 study groups (*P* = 0.046) (**b**). BMI: body mass index; BW: body weight; SE: standard error; SGLT2i: sodium–glucose cotransporter 2 inhibitors

Among a total of 10,237 patients with a paired BW data at baseline and follow-up period of around 12 weeks, there were 9228 (90.1%) patients having further follow-up visits (and BW data) beyond the follow-up date of BW. The median [25%, 75%] follow-up period with the last BW data available beyond the follow-up date of BW were 485 [252, 679] days. The log-term BW trajectories for the five study groups of different baseline BMI categories receiving SGLT2 treatment were summarized in Fig. 2a. The respective mean (SE) BW changes from baseline to the 3 months following-up in these 5 groups were − 0.67 (0.09), − 1.14 (0.07), − 1.23 (0.05), − 1.41 (0.07), and − 1.80 (0.07) kg. In general, the mean BW remained stable from 3 months onward across all study categories, and there is no difference of the BW slope across 5 study groups (*P* = 0.712) (Fig. 2a). The log-term BW trajectories for the three study groups of different BW-loss categories following SGLT2 treatment were summarized in Fig. 2b. The respective mean (SE) BW changes from baseline to the 3 months following-up in these 3 groups were − 1.36 (0.04), − 2.01 (0.01), and − 5.90 (0.08) kg. The mean (SE) BW slope from 3 months until the last available measurements among participants receiving SGLT2i was − 0.67 (− 0.50), − 0.14 (− 0.13), and 0.85 (0.23) kg/year in these 3 groups. There is significant difference of the BW slope across 3 study groups (*P* = 0.046) (Fig. 2b).

### ***Predictors of significant body weight loss of ***$\ge$*** 5%***

**Fig. 3:**
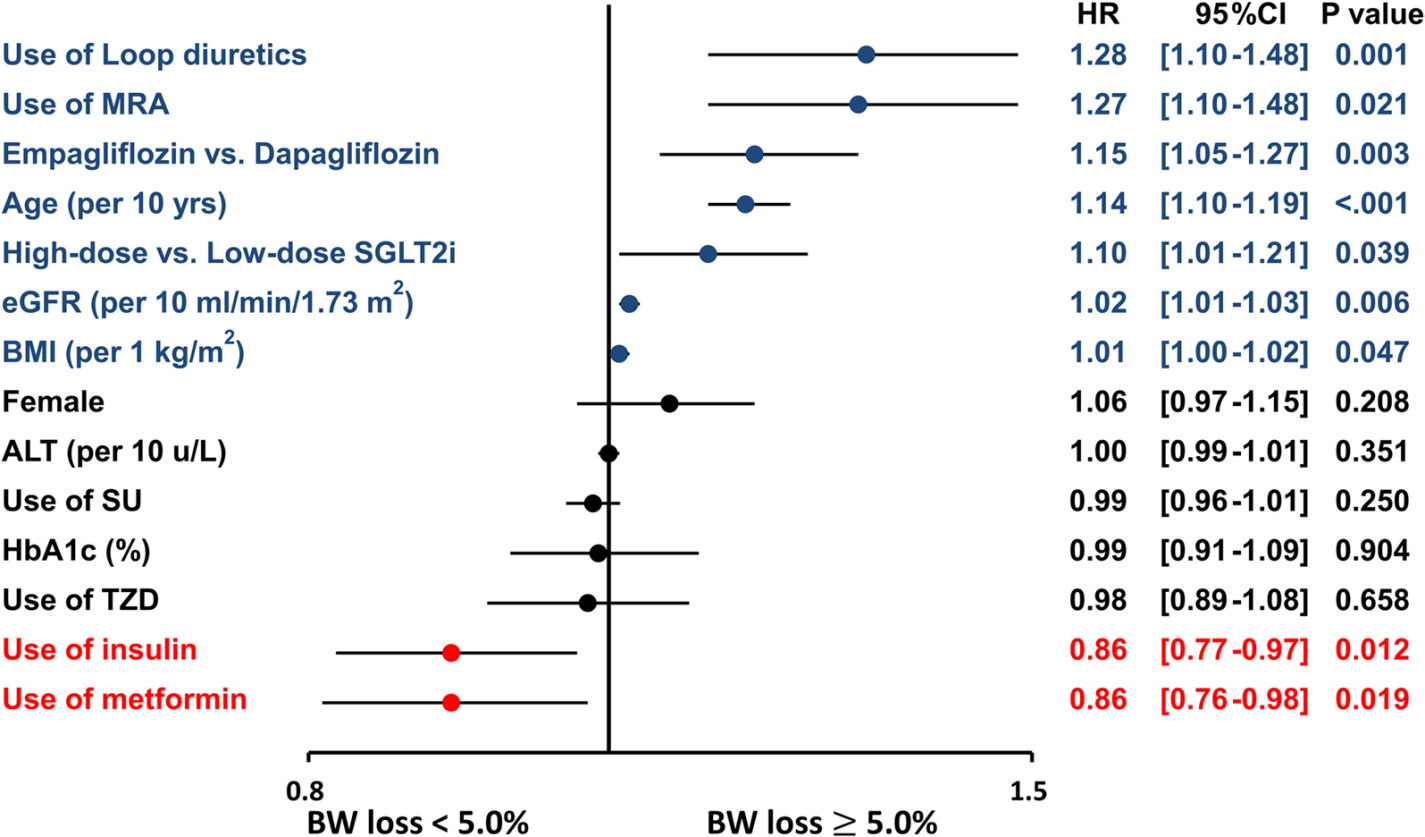
Factors associated with ≥ 5% BW loss in patients treated with SGLT2i. Multivariate analysis indicated that the use of diuretics, old age, a high-dose SGLT2i, use of empagliflozin in relative to dapagliflozin, higher estimated glomerular filtration rate, and higher BMI were independent factors associated with a BW loss of $\ge$ 5.0%, whereas use of insulin or metformin was independently associated with a lower risk of BW loss of $\ge$ 5.0% following SGLT2i initiation. ALT: aminotransferases; BMI: body mass index; BW: body weight; eGFR: estimated glomerular filtration rate; HbA1c: hemoglobin A1c; MRA: mineralocorticoid receptor antagonist; SGLT2i: sodium–glucose cotransporter 2 inhibitors; TZD: thiazolidinedione; AF: atrial fibrillation; T2DM: type 2 diabetes mellitus

Multivariate analysis indicated that the use of diuretics, old age, high-dose SGLT2i, use of empagliflozin rather than dapagliflozin, higher estimated glomerular filtration rate, and higher BMI were independent factors associated with a BW loss of $\ge$ 5.0%, whereas use of insulin or metformin was independently associated with a lower risk of BW loss of $\ge$ 5.0% following SGLT2i initiation (Fig. 3).

### Risk for new-onset AF with SGLT2i across study groups

**Fig. 4:**
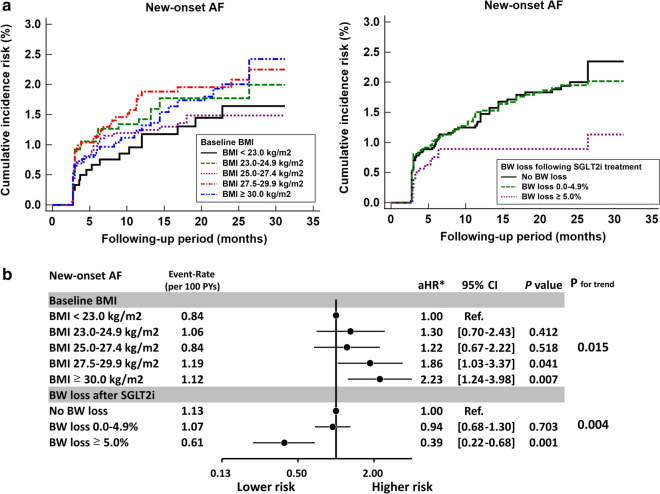
New-onset AF risk in patients with T2DM in different categories of baseline BMI and BW loss after SGLT2i treatment. Cumulative incidence risk of new-onset AF for T2DM patients in different categories of baseline BMI and BW loss following SGLT2i treatment. **a** Compared with those with a baseline BMI of < 23 kg/m^2^, AF risk significantly increased at baseline BMI $\ge$ 27.5 kg/m^2^ after multivariate adjustment (*P* for trend = 0.015). Compared with those without BW loss after 12 weeks of SGLT2i treatment, AF risk significantly decreased at a BW loss of $\ge$ 5.0% after multivariate adjustment (*P* for trend = 0.004). **b** *Risk of outcome was adjusted for age, sex, different SGLT2i drugs and dosage, baseline comorbidities as shown in Tables 1, 2, HbA1c, eGFR, and use of antiplatelet therapy, statin, angiotensin system inhibitor, and all anti-hypoglycemic agents. AF: atrial fibrillation; CI: confidence interval; eGFR: estimated glomerular filtration rate; HbA1c: glycated hemoglobin A1c; aHR: adjusted hazard ratio; SGLT2i: sodium–glucose cotransporter 2 inhibitor; T2DM: type 2 diabetes mellitus; BMI: body mass index; BW: body weight; SE: standard error; MRA: mineralocorticoid receptor antagonist; TZD: thiazolidinedione

Compared with those with a baseline BMI of < 23.0 kg/m^2^, AF risk significantly increased in patients with baseline BMI of $\ge$ 27.5 kg/m^2^ (adjusted hazard ratios [aHRs; 95% CIs]: 1.86 [1.03–3.37] for BMI of 27.5–29.9 kg/m^2^ and 2.23 [1.24–3.98] for BMI of $\ge$ 30.0 kg/m^2^; *P* for trend = 0.015) after multivariate adjustment of baseline characteristics. Compared with those without BW loss following SGLT2i treatment, AF risk significantly decreased at a BW loss of $\ge$ 5.0% (aHRs [95% CIs]: 0.39 [0.22–0.68]; *P* = 0.001) after multivariate adjustment of baseline characteristics (Fig. 4).

**Fig. 5:**
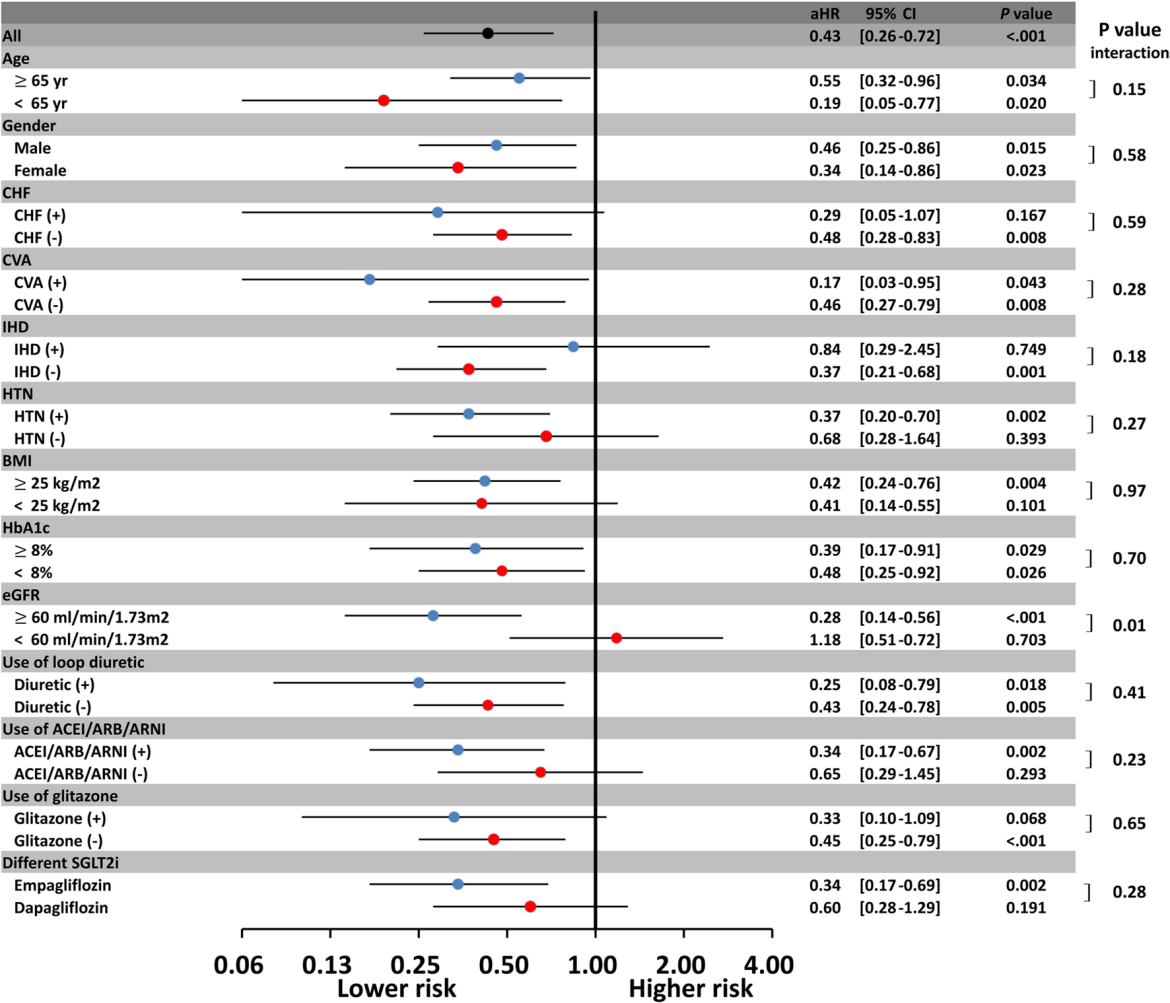
Subgroup analysis of risk of $\ge$ 5% BW loss on AF incidence in patients with T2DM treated with SGLT2i. The benefit of BW loss associated with SGLT2i treatment in the reduction of AF risk persisted across most T2DM subgroups, regardless of underlying comorbidities, baseline BMI, or different HbA1c. Notably, SGLT2i use was associated with greater reductions in new-onset AF events in patients with normal renal function than in those with impaired renal function (*P* for interaction = 0.01). ACEI: angiotensin-converting enzyme inhibitor; ARB: angiotensin receptor blocker; ARNI: angiotensin receptor-neprilysin inhibitor; BMI: body mass index; CHF: congestive heart failure; CVA: cerebral vascular disease; eGFR: estimated glomerular filtration rate; HbA1c: hemoglobin A1c; HTN: hypertension; IHD: ischemic heart disease AF: atrial fibrillation; CI: confidence interval; eGFR: estimated glomerular filtration rate; HbA1c: glycated hemoglobin A1c; aHR: adjusted hazard ratio; SGLT2i: sodium–glucose cotransporter 2 inhibitor; T2DM: type 2 diabetes mellitus; BMI: body mass index; BW: body weight; SE: standard error; MRA: mineralocorticoid receptor antagonist; TZD: thiazolidinedione

Subgroup analysis revealed that a $\ge$ 5% decrease in BW was associated with a lower new-onset AF risk than was a < 5% reduction in BW across most subgroups (*P* for interaction > 0.05; Fig. 5). Furthermore, SGLT2i use was associated with greater reductions in new-onset AF events in patients with normal renal function than in those with impaired renal function (*P* for interaction = 0.01).

### Risk for major adverse cardiovascular events/heart failure hospitalization with SGLT2i across study groups

**Fig. 6:**
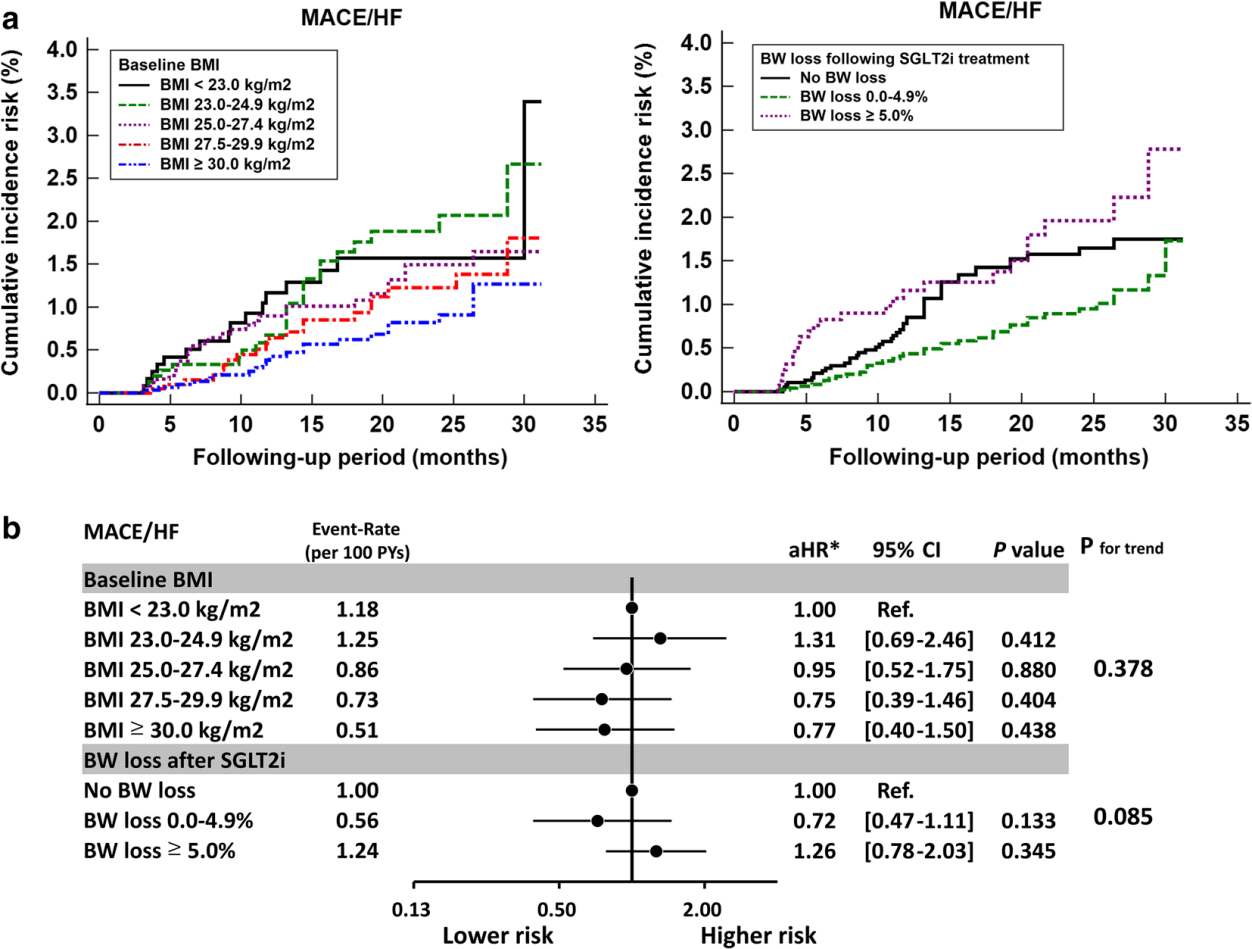
Major adverse cardiovascular event (MACE) or heart failure (HF) hospitalization risk in patients with T2DM in different categories of baseline BMI and BW loss following SGLT2i treatment. Cumulative incidence risk of MACE/HF for T2DM patients in different categories of baseline BMI and BW loss following SGLT2i treatment. **a** Neither baseline BMI nor posttreatment BW loss predicted the risk of MACE/HF hospitalization after multivariate adjustment (*P* for trend > 0.05). **b** *Risk of outcome was adjusted for age, sex, different SGLT2i drugs and dosage, baseline comorbidities as shown in Tables 1, 2, HbA1c, eGFR, and use of antiplatelet therapy, statin, angiotensin system inhibitor, and all anti-hypoglycemic agents. HF: heart failure; MACE: major adverse cardiovascular event AF: atrial fibrillation; CI: confidence interval; eGFR: estimated glomerular filtration rate; HbA1c: glycated hemoglobin A1c; aHR: adjusted hazard ratio; SGLT2i: sodium–glucose cotransporter 2 inhibitor; T2DM: type 2 diabetes mellitus; BMI: body mass index; BW: body weight; SE: standard error; MRA: mineralocorticoid receptor antagonist; TZD: thiazolidinedione

We also assessed the incidence of major adverse cardiovascular events (MACE) or heart failure (HF) hospitalization based on different baseline BMI and posttreatment BW loss categories. In contrast to the AF outcome, neither baseline BMI nor posttreatment BW loss predicted the risk of MACEor HF hospitalization after multivariate adjustment of baseline characteristics (*P* for trend > 0.05; Fig. 6).

## Discussion

This is the first study to investigate the impact of baseline BMI and amount of BW loss after SGLT2i treatment on the risk of new-onset AF in an Asian population with T2DM. The main findings of this study are as follows: (i) Baseline BMI $\ge$ 27.5 kg/m^2^ is independently associated with a significantly increased risk of new-onset AF in patients with T2DM treated with SGLT2i. (ii) A $\ge$ 5% reduction in BW after 12-week SGLT2i treatment is independently associated with a significantly lower new-onset AF risk in these patients. (iii) The benefit of a ≥ 5% BW loss associated with SGLT2i treatment in reducing AF risk persisted across all T2DM subgroups, irrespective of underlying comorbidities, baseline BMI, or DM status (*P* for interaction > 0.05). (iv) By contrast, neither baseline BMI nor posttreatment BW loss predicted the risk of MACE/HF in patients with T2DM treated with SGLT2i.

The presence of diabetes is a critical risk factor for cardiovascular events, heart failure hospitalization, and new-onset AF development [2, 3]. Overweight and obese patients have an increased new-onset AF risk [18]. Our findings are consistent with the limited studies exploring the relationship between BMI and AF in patients with diabetes. Grundvold et al. reported that baseline overweight (BMI = 25.0–29.9 kg/m^2^) and obesity (BMI $\ge$ 30.0 kg/m^2^) were associated with a 1.9-fold and 2.9-fold higher incident AF risk in 7169 patients with T2DM [19]. Kim et al. reported that overweight and obesity were significantly associated with an increased new-onset AF risk among 842,848 patients with diabetes, and diabetes severity had synergistic effects on new-onset AF risk [20]. Similarly, post hoc analysis of the ACCORD trial also indicated that obesity (BMI = 30.0–39.9 kg/m^2^) and severe obesity (BMI $\ge$ 30.0 kg/m^2^) are associated with an increased risk of AF in 10,074 patients with T2DM, but the interaction was significant only in men. Taken together, these findings imply that diabetes and obesity may have synergistic effects on incident AF risk, probably because excess adiposity–induced proinflammatory cascade and oxidative stress and diabetes-induced chronic hyperglycemia and glucose fluctuation are both implicated in AF pathogenesis [21–23].

SGLT2is inhibit glucose reabsorption from the proximal renal tubules, resulting in glycosuria [24], the magnitude of which is proportional to the plasma glucose level above the threshold [25]. Moreover, SGLT2is have cardioprotective and renoprotective effects in diabetic or nondiabetic patients through numerous mechanisms of action, independent of their glucose-lowering effect. Notably, post hoc analysis of the DECLARE-TIMI 58 trial and limited observational data reported a reduction in the risk of new-onset AF in patients with T2DM with/without established cardiovascular risk treated with SGLT2i versus those treated with the current standard-of-care antihypoglycemic agents [9, 10]. Although how SGLT2i reduces AF risk remains unclear, limited clinical and experimental studies have proposed a few explanations. At the cellular level, AF upregulates Na^+^/H^+^ exchanger (NHE). Empagliflozin and cariporide, a well‐described selective NHE inhibitor, both directly inhibit NHE in human atrial cardiomyocytes to a comparable extent [26]. NHE activation resulted in the accumulation of intracellular Na^+^, which reduces/reverses the driving force of the Na^+^/Ca^2+^ exchanger–mediated Ca^2+^ efflux, leading to intracellular Ca^2+^ overload and consequent atrial arrhythmia. Oxidative stress and inflammation were involved in the mechanisms of the promotion of electrical and structural substrates for AF [27]. Yurista et al. [28] demonstrated that SGLT2is can restore mitochondrial function, ameliorate electrical and structural remodeling, and prevent AF in high-fat diet– induced or streptozotocin-induced diabetic rats. Epicardial fat may infiltrate into the atrial myocardium, which could disrupt the depolarization wave front, favoring micro-re-entry circuits and causing local conduction block; this mechanism is also implicated in AF pathogenesis [29]. Some studies have reported that epicardial adipose tissue volume significantly decreased after 6-month SGLT2i treatment compared with the conventional treatment in patients with T2DM with coronary artery disease [30]. SGLT2is also reduced sympathetic overdrive, which plays a vital role in the development and maintenance of AF [31]. SGLT2is also exhibit some blood pressure-lowering effect and promote diuresis and natriuresis and hence could reduce atrial dilation and consequent atrial arrhythmia [32]. Obesity is an independent risk factor of incident AF, and BW loss following SGLT2i is thus proposed to be one of the important factors directly associated with the reduction of AF incidence. BW loss (either fat mass or free water loss) following SGLT2i treatment may be also associated with a reduction of blood pressure, reduced atrial dilation due to natriuresis and diuresis, and a reduction of sympathetic overactivity, which can partially explain how SGLT2i reduce the risk of AF. There were several protection mechanisms which may not be directly related to the effect of BW loss following SGLT2i treatment, including the reduction of epicardial fat, promotion of mitochondrial biogenesis, reduction of reactive oxygen species, inhibition of sodium–proton exchanger, improvement of hyperuricemia and hypomagnesemia, and improvement of insulin resistance, also suggested a few possible explanations for the anti-arrhythmic effect noted with SGLT2i [32].

Although obesity is known to be associated with the risk of new-onset incident AF, whether intentional BW loss can reduce AF risk remained uncertain. Studies have indicated that sustained BW loss dose-dependently reduced the burden of AF and symptom severity in patients with established AF [11, 13]. However, these studies might have been limited by overweight individuals with established AF. Moreover, whether BW loss can reduce new-onset AF risk in the general population without AF remains unclear. The Look AHEAD randomized controlled trial studied 5067 overweight or obese individuals with T2DM without prevalent AF. Although the intensive lifestyle intervention group achieved a mean percentage BW loss of 6.0% compared with 3.5% in the control group, no difference was noted in the risk for the risk of new-onset AF (hazard ratio [HR]: 0.99) [33]. Ball et al. reported that a decrease in BMI over time was associated with decreased AF risk and vice versa in 14,652 individuals over 10 years follow‐up [34]. Berkovitch et al. reported that each 1 kg/m^2^ reduction in BMI during follow-up was associated with a significant 7% reduction in the risk for the first attack of AF among 18,290 middle-aged adults [35]. Jamaly et al. reported that large amounts of BW loss through bariatric surgery reduced AF risk by 29% compared with usual care over long-term follow-up [36]. By contrast, Huxley et al. concluded that BW loss > 5% was associated with an increased AF risk in 14,219 participants from the ARIC study [37]. A recent meta-analysis of five pooled studies revealed that a 5% BW loss using a nonsurgical approach was not associated with a significant reduction in AF incidence (HR: 1.04) [38]. Whether intentional BW loss can diminish the risk of new-onset AF remains uncertain and may be related to the amount of BW loss, amount of overweight/obese population, and type of interventional approach [11, 38].

Thus far, only SGLT2is, glucagon-like peptide-1 (GLP1) receptor agonists, and metformin lead to BW loss, largely accounted for by body fat reduction [39], whereas other oral hypoglycemic agents and insulin have long been associated with BW gain in patients with T2DM [40, 41]. Most GLP1 agonists demonstrated cardiovascular benefit in the cardiovascular outcome trials [42–44]. GLP1 agonists also demonstrated multiple cardiovascular benefits, including an improvement of blood pressure and lipid profile; however, their use was associated with an increase of resting heart rate [45], possibly due to the augmented sympathetic nervous system activation directly mediated by GLP1 agonist [46]. Of note, previous meta-analysis indicated that patients treated with albiglutide was associated with a higher risk of AF or atrial flutter than all-comparators group [47], while other meta-analyses suggested that other GLP1 agonists were not associated with AF, with the only possible exception of albiglutide [48]. Therefore, whether GLP1 agonist could lead to a reduced risk of new-onset AF among patients with T2DM treated with/without SGLT2i remains unclear and requires further elucidation in the future. Our study is the first to report that BW loss associated with SGLT2i treatment was associated with a lower incident AF risk in patients with T2DM without AF. Notably, the benefit of significant BW loss associated with SGLT2i treatment in AF risk reduction persisted across most T2DM subgroups irrespective of baseline BMI, DM severity, and comorbidities. Further prospective and randomized research clarifying our results is warranted.

The multivariate analysis indicated that old age and a higher BMI at baseline were independent factors associated with a BW loss of $\ge$ 5.0% (Fig. 3). It is speculated that compensatory hyperphagia probably accounts for the partial off-set of BW loss mediated by SGLT2i [49]. Previous studies showed that younger diabetic patients complied worse with recommended diet and medication regimen for T2DM [50], which may partially explain why the cohort without BW reduction following SGLT2i treatment was significantly younger at baseline. It is also speculated that patients with a lower BMI at baseline tend to eat more to prevent further BW loss, in response to the urinary glucose excretion and associated caloric loss mediated by SGLT2i treatment. However, the causality between age or BMI and the diversity of BW loss following SGLT 2i treatment is unclear due to the retrospective and observational nature of the present study.

In contrast to the impact of new-onset AF, our study demonstrated that neither baseline BMI nor BW loss after SGLT2i treatment affected the risk of MACE/HF hospitalization in patients with T2DM treated with SGLT2i. This finding agreed with a recent analysis reporting that sustained BW loss was not associated with a lower risk of MACE/HF hospitalization among 12,521 prediabetes or T2DM participants from the ORIGIN trial [51]. The Look AHEAD trial applied a comprehensive lifestyle intervention program to overweight patients with type 2DM. Although an intensive lifestyle intervention focusing on BW loss resulted in greater reductions in glycated hemoglobin and greater initial improvements in all cardiovascular risk factors (except for low-density-lipoprotein cholesterol levels), it did not reduce the risk of cardiovascular events in the 5145 overweight or obese patients with T2DM [52]. Although the SGLT2i-meditated reduction in cardiovascular risk in patients with T2DM is often partially attributed to BW loss, our analysis indicated other mechanisms, including reductions in hyperglycemia, osmotic diuresis, reduced blood pressure, and renal protection, may play more crucial roles in the reduction of cardiovascular events in these patients [53].

## Study limitations

This study has several limitations. First, this was a retrospective and observational study. Several important parameters related to BW change [54], including physical activity or sedentary behavior, dietary behavior or component, use of alcohol or tobacco, and family history, were lacking in the present study. In addition, the BW check-up for each patient was not mandatory for each clinical visit time, which may cause a lack of BW data for some patients at specific time during their whole follow-up period. Furthermore, the clinical characteristics of the study patients were different across different categories of baseline BMI or posttreatment BW loss. Although we adjusted for several critical parameters relevant to clinical outcomes in the multivariate Cox regression models, some confounders were still probably present. Future prospective randomized studies are necessary to verify our findings. Second, the CGMH datasets is a closed medical system without external link to protect each patient’s privacy in CGMH database, which make us difficult to obtain the medical activity of each patient outside the CGMH database in Taiwan. The aforementioned limitation may have resulted in loss to follow-up or underestimation of medical activity of patients outside the CGMH system [55]. Third, the present study did not consider changes in the patients’ medical status or activity (e.g., new diagnosis of comorbidities, eGFR decline, and discontinuation or add-on of co-medication) during the follow-up period, which may affect the outcome. Finally, the database comprised an Asian population. In Asia, some obesity related comorbidities are similar or more prevalent in Asians than in Caucasians, even though Asians have lower prevalence of overweight and obesity than their Western counterparts [56]. In addition, the body composition and body fat distribution are different between Asian and Western populations [57]. Therefore, whether our results can be extrapolated to non-Asian population remains unclear.

## Conclusions

A baseline BMI of $\ge$ 27.5 kg/m^2^ and a $\ge$ 5% BW loss following SGLT2i treatment are independently associated with a significantly lower risk of new-onset AF in patients with T2DM treated with SGLT2i. The benefit of $\ge$ 5% BW loss following SGLT2i treatment in reduction of AF risk persisted across most T2DM subgroups, irrespective of underlying comorbidities, baseline BMI, or DM status. By contrast, neither baseline BMI nor BW loss after SGLT2i treatment were associated with major cardiovascular event or heart failure hospitalization risk in patients with T2DM treated with SGLT2i.

## Data Availability

The datasets used in this study were only available in the Chang Gung Medical Data Center, Taiwan. The SAS programs (codes) involved for this study are available from the corresponding author on reasonable request.

## Abbreviations

ACEI: Angiotensin-converting enzyme inhibitor
AF: Atrial fibrillation
ACEI: Angiotensin-converting enzyme inhibitor
ALT: Alanine aminotransferase;
ARB: Angiotensin receptor blocker
ARNI: Angiotensin receptor-neprilysin inhibitor
AMI: Acute myocardial infarction
APT: Antiplatelet agent
BMI: Body mass index
BW: Body weight
CKD: Chronic kidney disease
DBP: Diastolic blood pressure
DM: Diabetes mellitus
DPP4i: Dipeptidyl peptidase-4 inhibitor
eGFR: Estimated glomerular filtration rate
GLP1: Glucagon-like peptide-1
HbA1c: Hemoglobin A1c
HDL: High-density lipoprotein
IHD: Ischemic heart disease
LDL: Low-density lipoprotein
MRA: Mineralocorticoid Receptor Antagonist
PAD: Peripheral artery disease
SBP: Systolic blood pressure
SGLT2i: Sodium–glucose cotransporter-2 inhibitor
SU: Sulfonylurea
T2DM: Type 2 diabetes mellitus
